# Extracorporeal cardiopulmonary resuscitation for a patient awaiting heart transplantation: a case report

**DOI:** 10.31744/einstein_journal/2024RC0922

**Published:** 2024-10-21

**Authors:** Sávio Sérgio Ferreira Custódio, Isabela Argollo Ferreira, Carolina Cáfaro, Bruno de Arruda Bravim, Bárbara Rubim Alves, Gustavo Niankowski Saliba, Daniel Joelsons

**Affiliations:** 1 Hospital Israelita Albert Einstein São Paulo SP Brazil Hospital Israelita Albert Einstein, São Paulo, SP, Brazil.

**Keywords:** Cardiac arrest, Cardiopulmonary resuscitation, Extracorporeal membrane oxygenation, Heart transplantation

## Abstract

Extracorporeal cardiopulmonary resuscitation is a method of cardiopulmonary resuscitation that uses extracorporeal membrane oxygenation with conventional cardiopulmonary resuscitation and has emerged as a promising intervention for patients with refractory cardiac arrest. This report describes the case of a 44-year-old man at significant risk for mortality according to his high RESCUE-IHCA Score who was awaiting heart transplantation and experienced in-hospital cardiac arrest during hemodialysis. Prompt recognition, immediate activation of the extracorporeal membrane oxygenation team, and initiation of support within 60 minutes contributed to a favorable outcome. This case emphasizes important considerations associated with extracorporeal cardiopulmonary resuscitation implementation, including optimal patient selection, intervention timing, and nuances of cannulation procedures. Continuous monitoring, involvement of a specialized extracorporeal membrane oxygenation team, recognition of extracorporeal membrane oxygenation as bridge therapy, and integration of extracorporeal membrane oxygenation with definitive treatment strategies are highlighted. Extracorporeal cardiopulmonary resuscitation is a vital intervention for patients with refractory cardiac arrest, and adequate patient selection and swift implementation are crucial to improving outcomes. A comprehensive review of extracorporeal cardiopulmonary resuscitation is also presented to highlight its efficacy and challenges associated with in-hospital cardiac arrest.

## INTRODUCTION

**Figure 1:**
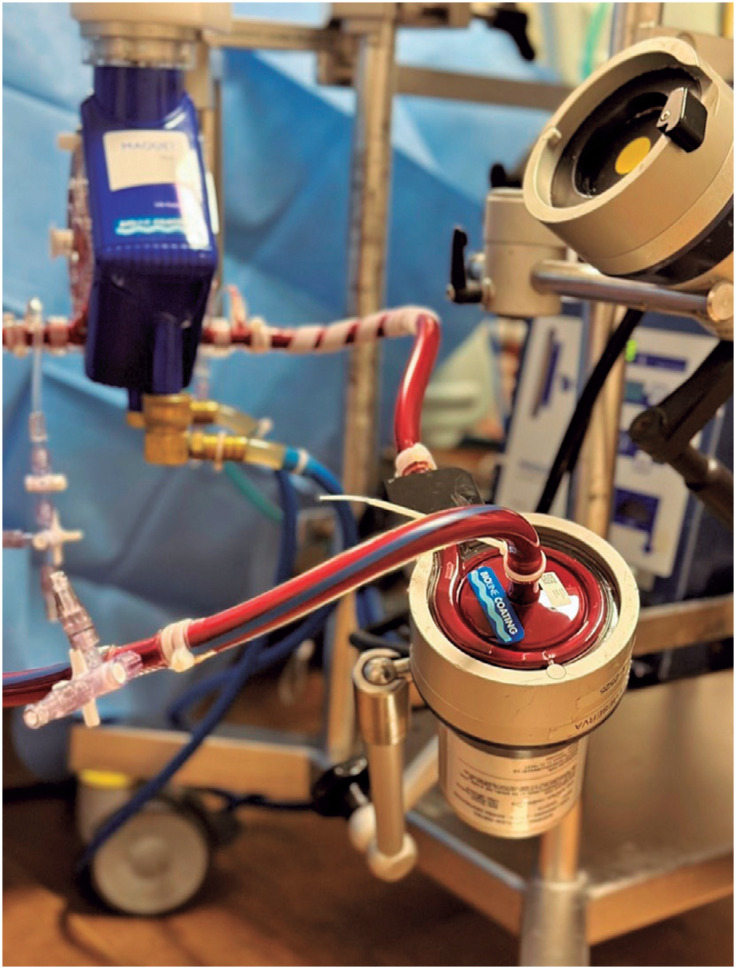
Extracorporeal membrane oxygenation pump and membrane by Getinge^®^

Source: authors’ personal digital archive.

Extracorporeal cardiopulmonary resuscitation (ECPR) is a method of cardiopulmonary resuscitation (CPR) that uses extracorporeal membrane oxygenation (ECMO) (Figure 1) with conventional CPR and is initiated during the period of chest compressions. Extracorporeal cardiopulmonary resuscitation can be used for both out-of-hospital cardiac arrest and in-hospital cardiac arrest (IHCA).^(1)^ Compared to the survival rates of conventional CPR, those of ECPR are higher, especially for patients who received conventional CPR for more than 10 minutes after witnessed IHCA and particularly for cases of cardiac origin.^(2)^

The implementation of ECPR for patients with IHCA poses a significant challenge because of the multiple comorbidities and inherent risks associated with this procedure. Determining the precise indications and contraindications for ECPR is crucial to optimizing patient outcomes. Observational studies have reported ECPR survival rates of 20% to 40% for patients with IHCA; however, additional randomized trials are necessary to verify its impact on survival.^(3)^ Successful ECPR depends on appropriate clinical indications and factors such as age, CPR duration and quality, comorbidities, initial cardiac rhythm, and time since cardiac arrest. The International Liaison Committee on Resuscitation has suggested that ECPR may be considered as a rescue therapy for select patients with IHCA when conventional CPR fails to restore spontaneous circulation in settings where it can be implemented (weak recommendation, very low-certainty evidence).^(4)^

This report describes the clinical case of a patient awaiting heart transplantation who experienced IHCA during hemodialysis. The patient underwent ECPR and recovered his previous functionality despite the presence of factors associated with high morbidity and mortality as indicated by his high RESCUE-IHCA Score.^(3)^

## CASE REPORT

A 44-year-old man was hospitalized because of restrictive cardiomyopathy progressing to heart failure according to the Interagency Registry for Mechanically Assisted Circulatory Support (profile 3). He was awaiting heart transplantation and experienced cardiac arrest during hemodialysis. A "code blue" was promptly triggered, and advanced cardiac life support-guided CPR was initiated. After 34 minutes of unsuccessful CPR and continuous ventricular fibrillation without the return of spontaneous circulation, the ECMO team was activated and ECPR was indicated. The right femoral vein and left femoral artery were cannulated using the Seldinger technique. Venoarterial ECMO support was initiated 54 minutes after cardiac arrest. Subsequently, electrical stability was achieved without any further arrhythmias.

The patient was moved to the intensive care unit, where he experienced pulseless ventricular tachycardia that was treated with defibrillation and lidocaine 0.02mg/kg/min infusion. Extubation was performed after 5 days. No neurological deficits (Glasgow outcome scale score, 15) were observed, and the patient remained clinically stable with continuous inotropic support, the use of an intra-aortic balloon pump (1:1 mode), and venoarterial ECMO. At 13 days after cardiac arrest, successful heart transplantation and ECMO decannulation were performed. He was discharged from the intensive care unit 8 days after transplantation. His cerebral performance category score was 1 at the time of hospital discharge. This case report was approved by the Research Ethics Committee of *Hospital Israelita Albert Einstein* (CAAE: 72890223.4.0000.0071; #6.247.207).

## DISCUSSION

According to the Extracorporeal Life Support Organization database, ECPR, which is internationally acclaimed and used at global reference centers, has been performed for more than 9.500 patients during the past 5 years, resulting in a survival rate of 32%. However, robust data regarding prognostic factors, management strategies, and clinical outcomes are lacking, and there have been no randomized trials of ECPR for IHCA. Therefore, it is crucial to emphasize and integrate factors that have been consistently associated with improved outcomes in clinical practice.^(1)^

Experts agree that the following patients are likely to experience optimal outcomes: those younger than 70 to 75 years; those with a shockable rhythm during cardiac arrest; those with less than 5 minutes between cardiac arrest and CPR initiation; and those with less than 60 minutes between cardiac arrest and commencement of ECMO support.^(2)^

**Figure 2:**
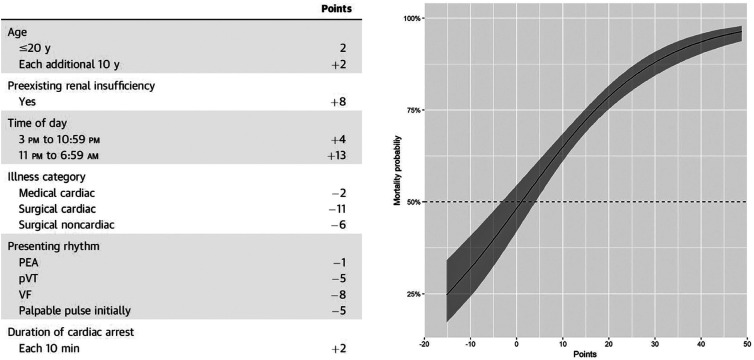
RESCUE-IHCA Score calculation and the predicted probability of death according to the RESCUE-IHCA Score The curve with a shaded 95% confidence interval shows the association between the score and in-hospital mortality among the study population.

PEA: pulseless electrical activity; pVT: pulseless ventricular tachycardia; VF: ventricular fibrillation.

Source: Tonna JE, Selzman CH, Girotra S, Presson AP, Thiagarajan RR, Becker LB, Zhang C, Rycus P, Keenan HT; American Heart Association Get With the Guidelines-Resuscitation Investigators. Resuscitation Using ECPR During In-Hospital Cardiac Arrest (RESCUE-IHCA) Mortality Prediction Score and External Validation. JACC Cardiovasc Interv. 2022;15(3):237-47.^(3)^

The RESCUE-IHCA study by Tonna et al., which included 1075 patients treated with ECPR, reported a hospital discharge survival rate of 28% and identified the following factors associated with in-hospital death: age, time of day, initial rhythm, history of renal failure, patient type (cardiac/noncardiac or clinical/surgical), and cardiac arrest duration. These factors comprise the statistically robust RESCUE-IHCA Score, which can effectively predict in-hospital mortality of patients who receive ECPR and serves as a real-time prognostic tool for physicians (Figure 2).^(3)^ Our patient had a RESCUE-IHCA Score of 18, indicating a 75% probability of in-hospital mortality.

The optimal timing of ECPR during CPR has not yet been determined. Early cannulation provides prompt circulatory support but is associated with procedural risks. Furthermore, some patients may regain spontaneous circulation with advanced cardiac life support alone. A balanced approach suggests 10 to 20 minutes of high-quality CPR, with 20 minutes recommended for shockable rhythms and 10 minutes recommended for non-shockable rhythms. The CPR duration before ECPR for our patient was prolonged to 34 minutes.^(5,6)^

Despite multiple comorbidities, a high RESCUE-IHCA Score, and substantial mortality risk, our patient experienced a successful outcome, which was likely attributable to the rapid recognition of and immediate response to cardiac arrest, swift activation of the ECMO team, and initiation of support within 60 minutes of cardiac arrest.

Common exclusion criteria for ECPR include cardiac arrest of probable non-cardiac origin, current or recent ischemic or hemorrhagic stroke, history of severe organ dysfunction, and advance directives defining support limitations.^(1,7)^

The cannulation phase of ECPR is technically demanding and requires a highly skilled team. Studies have emphasized the importance of speed and reported that survival rates decreased by 4% to 25% for each 10-minute delay beyond the initial 10 to 20 minutes before ECMO initiation.

The usefulness of a distal limb reperfusion cannula for ECPR is unclear because it prevents limb ischemia from cannulation but complicates an emergency procedure that is already demanding. During ECPR for our patient, a small-caliber cannula was intentionally chosen to avoid the use of a distal limb reperfusion cannula. Although this approach reduces procedural complexity, vigilant monitoring of limb perfusion is required after cannulation.

After extracorporeal support is established, specific hemodynamic parameters must be tracked, support levels must be adjusted, and complications must be monitored by a specialized ECMO team. Constant surveillance and reassessments by multiple professionals are imperative to achieving successful clinical outcomes.^(1,8,9)^

Although effective ECMO management has a critical role in clinical outcomes, it largely functions as a bridge therapy. Therefore, its integration with a definitive treatment strategy is crucial.

## CONCLUSION

Extracorporeal cardiopulmonary resuscitation is vital for patients with refractory cardiac arrest who are unresponsive to standard cardiopulmonary resuscitation. Appropriate patient selection is the key to survival. Despite the poor initial prognosis (RESCUE-IHCA Score, 18) of our patient, high-quality cardiopulmonary resuscitation initiation and extracorporeal membrane oxygenation within 60 minutes resulted in a successful outcome. Additional studies are necessary to verify the effect of extracorporeal cardiopulmonary resuscitation on survival.
